# Tiotropium Respimat® in asthma: a double-blind, randomised, dose-ranging study in adult patients with moderate asthma

**DOI:** 10.1186/1465-9921-15-61

**Published:** 2014-06-03

**Authors:** Kai-Michael Beeh, Petra Moroni-Zentgraf, Othmar Ablinger, Zuzana Hollaenderova, Anna Unseld, Michael Engel, Stephanie Korn

**Affiliations:** 1 insaf Respiratory Research Institute GmbH, Wiesbaden, Germany; 2 Boehringer Ingelheim Pharma GmbH & Co. KG, Ingelheim am Rhein, Germany; 3 Thalheim bei Wels, Austria; 4 Boehringer Ingelheim RCV GmbH & Co. KG, Vienna, Austria; 5 Boehringer Ingelheim Pharma GmbH & Co. KG, Biberach an der Riss, Germany; 6 Pulmonary Department, Mainz University Hospital, Johannes Gutenberg University, Mainz, Germany

**Keywords:** Asthma, Tiotropium, Respimat, Cholinergic antagonists, Bronchodilator agents, Clinical trial

## Abstract

**Background:**

Tiotropium, a once-daily long-acting anticholinergic bronchodilator, when administered via Respimat® SoftMist™ inhaler (tiotropium Respimat®) significantly reduces the risk of severe exacerbations and improves lung function in patients with severe persistent asthma that is not fully controlled despite using inhaled corticosteroids (ICS) and long-acting β_2_-agonists. To further explore the dose–response curve in asthma, we investigated the efficacy and safety of three different doses of tiotropium Respimat® as add-on to ICS in symptomatic patients with moderate persistent asthma.

**Methods:**

In this randomised, double-blind, placebo-controlled, four-way crossover study, patients were randomised to tiotropium Respimat® 5 μg, 2.5 μg or 1.25 μg or placebo Respimat®, once daily in the evening. Each treatment was administered for 4 weeks, without washout between treatment periods. Eligibility criteria included ≥60% and ≤90% of predicted normal forced expiratory volume in 1 second (FEV_1_) and seven-question Asthma Control Questionnaire mean score of ≥1.5. Patients were required to continue maintenance treatment with stable medium-dose ICS for at least 4 weeks prior to and during the treatment period. Long-acting β_2_-agonists were not permitted during the treatment phase. The primary efficacy end point was peak FEV_1_ measured within 3 hours after dosing (peak FEV_1(0-3h)_) at the end of each 4-week period, analysed as a response (change from study baseline).

**Results:**

In total, 149 patients were randomised and 141 completed the study. Statistically significant improvements in peak FEV_1(0-3h)_ response were observed with each tiotropium Respimat® dose versus placebo (all P < 0.0001). The largest difference from placebo was with tiotropium Respimat® 5 μg (188 mL). Trough FEV_1_ and FEV_1_ area under the curve (AUC)_(0-3h)_ responses were greater with each tiotropium Respimat® dose than with placebo (all P < 0.0001), and both were greatest with 5 μg. Peak forced vital capacity (FVC)_(0-3h)_, trough FVC and FVC AUC_(0-3h)_ responses, versus placebo, were greatest with tiotropium Respimat® 5 μg (P < 0.0001, P = 0.0012 and P < 0.0001, respectively). Incidence of adverse events was comparable between placebo and all tiotropium Respimat® groups.

**Conclusions:**

Once-daily tiotropium Respimat® add-on to medium-dose ICS improves lung function in symptomatic patients with moderate asthma. Overall, improvements were largest with tiotropium Respimat® 5 μg.

**Trial registration:**

ClinicalTrials.gov identifier NCT01233284.

## Background

Currently, inhaled corticosteroids (ICS) are the de facto first-line therapy for the management of poorly controlled, persistent asthma. In accordance with the Global Initiative for Asthma guidelines [1], for patients with moderate asthma who remain symptomatic despite using ICS, therapy is typically ‘stepped up’ by increasing the ICS dose and/or adding a long-acting β_2_-agonist (LABA) to the maintenance treatment regimen. Leukotriene modifiers or sustained-release theophylline, added on to low-dose ICS, are alternative options for these patients. Despite this range of therapeutic choices, at least 40% of patients with asthma have poorly controlled disease [2-4]. Further, in patients who fail to gain control with an ICS + LABA fixed-dose combination, the remaining options, such as further upwards titration of ICS, systemic glucocorticosteroids and anti-immunoglobulin E therapy, may have several limitations with respect to side effects, risk:benefit ratio, convenience and efficacy [1,5,6].

Tiotropium, a once-daily long-acting anticholinergic bronchodilator, is the established first-line maintenance bronchodilator for the management of chronic obstructive pulmonary disease [7,8]. Recently, a number of clinical trials have investigated tiotropium as add-on to at least ICS for the long-term control of asthma, across a range of severities. In the investigator-led TALC trial (Tiotropium Bromide as an Alternative to Increased Inhaled Glucocorticoid in Patients Inadequately Controlled on a Lower Dose of Inhaled Corticosteroid; three-way crossover, 14 weeks per treatment, 210 patients), improvements in lung function in patients with asthma treated with tiotropium (via HandiHaler®; Boehringer Ingelheim Pharma GmbH & Co. KG, Ingelheim am Rhein, Germany) plus beclomethasone (two puffs of 40 μg, twice daily) were shown to be superior to a doubling of the ICS dose and similar to the addition of salmeterol [9]. In a 16-week proof-of-concept trial in B16-Arg/Arg patients with symptomatic moderate asthma already receiving ICS, tiotropium 5 μg (administered via the Respimat® SoftMist™ inhaler [hereinafter referred to as tiotropium Respimat®; Boehringer Ingelheim Pharma GmbH & Co. KG]) was superior to placebo Respimat® and non-inferior to salmeterol [10]. A second proof-of-concept study (three-way crossover, 8 weeks per treatment) in patients with more severe disease – severe persistent asthma, and receiving ICS + LABA – demonstrated that lung function improved significantly with tiotropium Respimat® 10 μg or 5 μg [11]. Slightly higher peak forced expiratory volume in 1 second (FEV_1_) responses were observed in the 10 μg arm [11], and there were statistically significant improvements in pre-dose morning and evening peak expiratory flow (PEF_am_ and PEF_pm_) with 10 μg versus 5 μg. Kerstjens and colleagues subsequently reported results from two long-term, replicate, 1-year Phase III trials of tiotropium Respimat® 5 μg, also in patients with symptomatic severe asthma despite using ICS + LABA. In this cohort of 912 patients, tiotropium Respimat® 5 μg, administered as add-on to ICS + LABA, significantly reduced the risk of severe exacerbations and improved lung function, compared with placebo Respimat® as add-on to ICS + LABA [12].

In light of the findings with the 10 μg and 5 μg doses of tiotropium Respimat® (outlined above), the aim of the present study was to further explore the dose–response curve by comparing the efficacy and tolerability of once-daily evening dosing of tiotropium Respimat® 5 μg, 2.5 μg or 1.25 μg, versus placebo Respimat®, each added on to medium-dose ICS, in adult patients with symptomatic moderate asthma.

## Methods

This was a Phase II, randomised, double-blind, placebo-controlled, crossover study to investigate the efficacy and safety of three doses of once-daily tiotropium Respimat®. The study was conducted in 19 sites in three European countries (Germany, Austria and Ukraine; ClinicalTrials.gov identifier NCT01233284), and was carried out in accordance with the principles of the Declaration of Helsinki and the International Conference on Harmonisation Good Clinical Practice Guidelines. All patients provided written, informed consent.

### Study design

**Figure 1:**
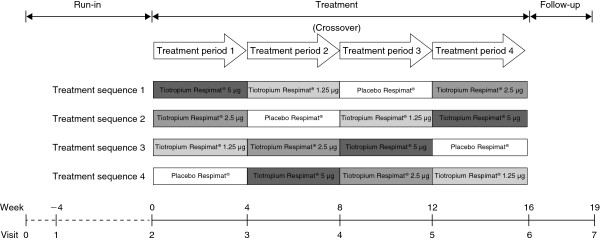
Study design.

After an initial screening visit and a 4-week run-in period, patients were randomised to one of four treatment sequences, during which they received each of the four treatments (tiotropium 5 μg, 2.5 μg or 1.25 μg or placebo, all delivered via the Respimat® SoftMist™ inhaler) (Figure 1). Each treatment was administered for 4 weeks, and there was no washout between treatment periods as pharmacodynamic steady state with tiotropium is known to be achieved after 3 weeks in chronic obstructive pulmonary disease [13,14]. Seven clinic visits were scheduled: at screening (Visit 0); prior to the 4-week run-in (Visit 1); at randomisation (after the 4-week run-in [Visit 2]); every 4 weeks at the end of each treatment period (Visits 3–6); and 21 days following the end of the final treatment period (Visit 7).

Tiotropium Respimat® (two puffs) or placebo Respimat® (two puffs) was administered once daily in the evening between 18:00 and 20:00. All patients were required to continue maintenance treatment with stable medium-dose ICS (400–800 μg budesonide or equivalent) for at least 4 weeks prior to Visit 1 and until Visit 7. Patients using ICS plus short-acting β_2_-agonist or ICS + LABA fixed-dose combinations were switched to the same dose of ICS mono-product at least 8 or 24 hours, respectively, prior to Visit 1. Study medication was to be taken immediately after ICS inhalation if normal ICS dosing was in the evening. Concomitant use of the following was not permitted for maintenance treatment: systemic oral or depot corticosteroids; anticholinergics other than tiotropium; LABAs; ICS plus short-acting β_2_-agonist or ICS + LABA fixed-dose combinations; leukotriene modifiers; anti-immunoglobulin E treatment; chromone; methylxanthines; and phosphodiesterase-4 inhibitors. Salbutamol hydrofluoroalkane metered-dose inhaler was provided by the sponsor as rescue medication for use as needed. Permitted medication for the treatment of acute asthma exacerbations included salbutamol hydrofluoroalkane metered-dose inhaler, systemic corticosteroids and short-acting theophylline preparations.

#### Randomisation

Each patient received all treatments. Eligible patients were randomly allocated to one of the four treatment sequences at Visit 2 (Figure 1). The randomisation list was generated by Boehringer Ingelheim Pharma GmbH & Co. KG, Biberach an der Riss, Germany, using a validated pseudo-random number generator and a supplied seed number. A fixed block randomisation, with a block size of 4, was used to ensure balanced and equal assignment.

### Study patients

Male or female patients aged 18–75 years, with at least a 3-month history of asthma at the time of enrolment and an initial diagnosis of asthma made before the age of 40 years, were included in the study. Patients were required to have been on maintenance treatment with stable medium-dose ICS (400–800 μg budesonide or equivalent), alone or in a fixed-dose combination with a LABA or short-acting β_2_-agonist, for at least 4 weeks prior to Visit 1. A diagnosis of asthma confirmed at Visit 1 was required, with bronchodilator reversibility (15–30 minutes after 400 μg salbutamol) of ≥12% and ≥200 mL. Patients were required to have a seven-question Asthma Control Questionnaire (ACQ-7) mean score of ≥1.5 at Visits 1 and 2, to have a pre-bronchodilator FEV_1_ of ≥60% and ≤90% of predicted normal FEV_1_ at Visit 1, and to demonstrate absolute FEV_1_ variability within 30% between Visits 1 and 2. Patients were required never to have smoked, or to be ex-smokers who had stopped smoking at least 1 year prior to enrolment and had a smoking history of less than 10 pack-years. Patients were excluded for any of the following reasons: a diagnosis of chronic obstructive pulmonary disease or any respiratory disease other than asthma; myocardial infarction within the last 6 months; hospitalisation due to cardiac failure or unstable cardiac arrhythmia within the past year; treatment with anti-immunoglobulin E antibodies within 6 months prior to Visit 1.

### Study end points

All end points were determined at the end of each 4-week treatment period (Visits 3–6) and analysed as a response defined as change from study baseline (pre-treatment value measured at Visit 2 in the evening). The primary efficacy end point was peak FEV_1_ measured within the first 3 hours after dosing (peak FEV_1(0-3h)_). The following secondary efficacy end points were investigated: trough FEV_1_; peak forced vital capacity (FVC) within the first 3 hours after dosing (FVC)_(0-3h)_; trough FVC; FEV_1_ area under the curve (AUC) within the first 3 hours after dosing (FEV_1_ AUC_(0-3h)_); FVC AUC_(0-3h)_; pre-dose PEF_am_ and PEF_pm_ using the Asthma Monitor2+ device (AM2+®; ERT, Philadelphia, Pennsylvania, USA) based on the mean of the final week of each treatment period. Control of asthma, as assessed by ACQ-7 self-administered at the end of each 4-week treatment period, was an additional exploratory end point. Safety and tolerability were assessed based on the incidence and intensity of adverse events, and on changes in vital signs.

### Study assessments

Spirometric lung function tests were conducted at all in-clinic visits (Visits 1–6). Pre-dose lung function tests were scheduled between 18:00 and 20:00. At Visits 2–6, lung function tests were performed 10 minutes prior to and up to 3 hours after dosing of study treatment. ACQ-7 was self-administered at Visits 1–6 prior to lung function tests. Measurement of PEF_am_ and PEF_pm_ was to be performed prior to ICS and study treatment inhalation, at approximately the same time each day, from 06:00 to 08:00 for PEF_am_ and from 18:00 to 20:00 for PEF_pm_. Adverse events and concomitant medications were recorded on the electronic case report form at each visit. Vital signs were measured and recorded in conjunction with lung function tests at Visits 2–6.

### Statistical analyses

Efficacy data are reported for the full analysis set, which was defined as all randomised patients who were treated with at least one dose of study medication, had baseline data and had at least one on-treatment efficacy measurement after 4 weeks of treatment within a period. Data are presented as adjusted mean change from baseline after 4 weeks of treatment (defined as response), unless noted otherwise.

Evaluation of safety and tolerability was performed on the treated set, defined as all randomised patients who received at least one dose of study medication. Analysis of adverse events and vital signs was descriptive in nature.

To control the probability of a type I error in the primary efficacy analysis, stepwise testing of the null hypothesis was used to test the efficacy of tiotropium Respimat® 5 μg, then 2.5 μg and then 1.25 μg, each over placebo Respimat®. If the previous step was not successful, analysis of the current step was to be considered descriptive. To detect a treatment difference of 80 mL for peak FEV_1(0-3h)_ with 90% power, and assuming a standard deviation of 228 mL, it was calculated that 88 completed patients were required. Testing was performed with α = 0.025 (one-sided).

The pre-specified hypotheses were tested using a mixed effects model with repeated measures. The statistical model included ‘treatment’ and ‘period’ as fixed effects and ‘patient’ as a random effect. Study baseline, defined as pre-treatment values measured at Visit 2 in the evening, was included as covariate.

## Results

### Baseline demographics and disposition

**Figure 2:**
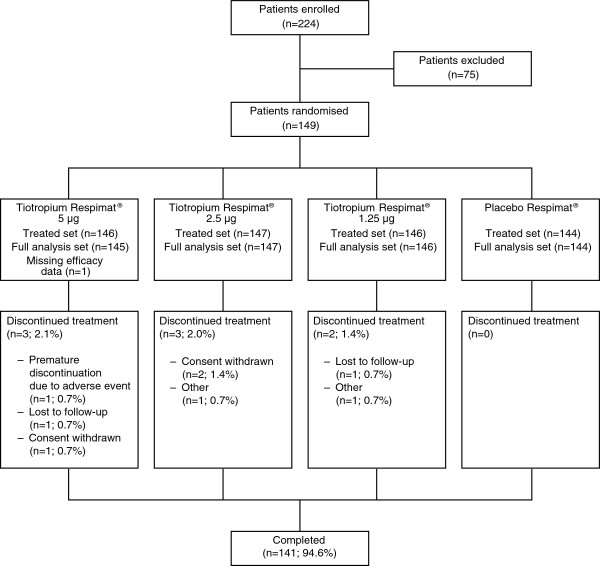
Patient disposition.

A total of 149 patients were randomised to the study, and 141 patients completed the study. The treated set comprised all 149 patients and the full analysis set comprised 148 patients (one patient had missing efficacy data and was lost to follow-up after Visit 2) (Figure 2). In the treated set, slightly more patients were female (55.0%) than male (45.0%), mean age was 49.3 years and mean body mass index was 26.9 kg/m^2^ (Table 1). Of the small proportion of patients who had previously smoked (19.5%; n = 29), the mean number of pack-years was 5.6. The remainder of the study population had never smoked (80.5%; n = 120) (Table 1).

**Table 1:** Baseline and demographic characteristics (treated set)

	**Total**
	**(n = 149)**
Age, years^a^	49.3 ± 13.3
Gender, n (%)	
Male	67 (45.0)
Female	82 (55.0)
Body mass index, kg/m^2 a^	26.9 ± 4.3
Smoking status, n (%)	
Current smoker	0
Ex-smoker	29 (19.5)
Never smoked	120 (80.5)
Smoking history, pack-years^a^	5.6 ± 2.5
Duration of asthma, years^a^	23.8 ± 13.4
FEV_1_^a^	
% of predicted value pre-bronchodilation at screening^b^	71.3 ± 7.1
% of predicted value post-bronchodilation at screening^b^	87.4 ± 10.2
Reversibility, L^b^	0.500 ± 0.239
% reversibility^b^	22.8 ± 10.2
Pre-dose at study baseline, L^c^	2.306 ± 0.689
FVC, pre-dose at baseline^c^, L^a^	3.639 ± 0.978
FEV_1_/FVC ratio at baseline^c^, %^a^	63.7 ± 10.1
ICS dose of stable maintenance treatment, μg^a,d^	659.2 ± 249.4

^a^Values are mean ± standard deviation; ^b^Visit 1; ^c^Measured 10 minutes prior to inhalation of study medication at Visit 2 (at randomisation); ^d^Budesonide equipotent dose. FEV_1_, forced expiratory volume in 1 second; FVC, forced vital capacity; ICS, inhaled corticosteroids.

### Efficacy

#### Primary analysis

**Figure 3:**
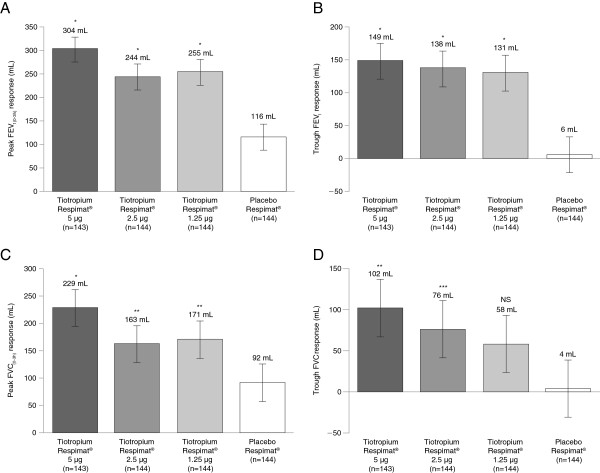
**Adjusted mean differences in lung function responses. (A)** Peak FEV_1(0-3h)_ response; **(B)** Trough FEV_1_ response; **(C)** Peak FVC_(0-3h)_ response; **(D)** Trough FVC response. Response defined as change from study baseline (pre-treatment value measured at Visit 2 in the evening). Adjusted mean difference from placebo Respimat^®^: *P < 0.001; **P < 0.01; ***P < 0.05. Bars: standard error. FEV_1_, forced expiratory volume in 1 second; FVC, forced vital capacity; NS, not significant; peak FEV_1(0-3h)_, peak forced expiratory volume in 1 second measured within the first 3 hours after dosing; peak FVC_(0-3h)_, peak forced vital capacity measured within the first 3 hours after dosing.

The addition of tiotropium Respimat® 5 μg, 2.5 μg or 1.25 μg to stable medium-dose ICS therapy was associated with improved lung function: at the end of the 4-week treatment period, statistically significant differences from placebo Respimat® in adjusted mean peak FEV_1(0-3h)_ responses were observed for all doses of tiotropium Respimat® (P < 0.0001 at all doses) (Figure 3). The largest adjusted mean difference from placebo Respimat® was observed with tiotropium Respimat® 5 μg (188 mL, 95% confidence interval: 140, 236).

#### Secondary analyses

Trough FEV_1_, FEV_1_ AUC_(0-3h)_, peak FVC_(0-3h)_, trough FVC and FVC AUC_(0-3h)_ responses with all doses of tiotropium Respimat® were larger than the responses observed with placebo Respimat®, and all were statistically significant except for trough FVC in the 1.25 μg group (Figure 3, Additional file 1: Table S1). Responses for each of these end points were all largest in the 5 μg group (Figure 3, Additional file 1: Table S1).

Exploratory analysis of the difference in response between tiotropium Respimat® doses found that peak FEV_1(0-3h)_, FEV_1_ AUC_(0-3h)_, peak FVC_(0-3h)_ and FVC AUC_(0-3h)_ responses were all statistically significantly greater with tiotropium Respimat® 5 μg than with either of the two lower doses (Table 2).

**Table 2:** Adjusted mean differences in lung function responses between tiotropium Respimat® doses

**Response parameter**	**Adjusted mean differences between treatments (95% CI), mL**		
	**Tiotropium Respimat® 5 μg versus tiotropium Respimat® 1.25 μg**	**Tiotropium Respimat® 5 μg versus tiotropium Respimat® 2.5 μg**	**Tiotropium Respimat® 2.5 μg versus tiotropium Respimat® 1.25 μg**
FEV_1_			
Peak FEV_1(0-3h)_	50 (2, 98)	60 (12, 108)	-10 (-58, 38)
Trough FEV_1_	18 (-30, 66)	11 (-36, 59)	7 (-41, 54)
FEV_1_ AUC_(0-3h)_	49 (3, 95)	51 (5, 97)	-2 (-48, 44)
FVC			
Peak FVC_(0-3h)_	58 (5, 111)	67 (14, 120)	-9 (-61, 44)
Trough FVC	44 (-15, 103)	27 (-33, 86)	17 (-42, 77)
FVC AUC_(0-3h)_	74 (22, 0.125)	63 (11, 114)	11 (-40, 62)

AUC, area under the curve; CI, confidence interval; FEV_1_, forced expiratory volume in 1 second; FEV_1_ AUC_(0-3h)_, forced expiratory volume in 1 second area under the curve measured within the first 3 hours after dosing; FVC, forced vital capacity; FVC AUC_(0-3h)_, forced vital capacity area under the curve measured within the first 3 hours after dosing; peak FEV_1(0-3h)_, peak forced expiratory volume in 1 second measured within the first 3 hours after dosing; peak FVC_(0-3h)_, peak forced vital capacity measured within the first 3 hours after dosing.

At the end of each 4-week treatment period, there was a statistically significant improvement in ACQ-7 score with all three tiotropium Respimat® doses (Additional file 1: Table S1).

Higher mean pre-dose PEF_am_ responses (measured with the AM2+® device) were observed with all three tiotropium Respimat® treatments compared with placebo Respimat® (difference from placebo: 5 μg, 20.846 L/min; 2.5 μg, 17.895 L/min; 1.25 μg, 18.550 L/min; all P < 0.0001). Higher mean pre-dose PEF_pm_ responses were also observed with all three tiotropium Respimat® treatments compared with placebo Respimat® (difference from placebo: 5 μg, 21.581 L/min; 2.5 μg, 14.577 L/min; 1.25 μg, 21.251 L/min; all P < 0.0001). No significant differences in PEF_am_ or PEF_pm_ responses were observed between the different tiotropium Respimat® doses.

### Safety and tolerability

Overall incidence of adverse events was comparable between placebo Respimat® and the three tiotropium Respimat® treatment doses (Table 3). Serious adverse events were reported for two patients (in the tiotropium Respimat® 5 μg group). Neither was considered to be drug-related. One patient was reported with alcohol abuse and panic attack, which led to hospitalisation. Both of this patient’s adverse events accounted for discontinuation of the study drug (the only case of discontinuation during the trial). The other patient was reported with inguinal hernia, which led to hospitalisation.

**Table 3:** Summary of adverse events in the treated set

**n (%)**	**Tiotropium Respimat® 5 μg**^**a**^	**Tiotropium Respimat® 2.5 μg**^**a**^	**Tiotropium Respimat® 1.25 μg**^**a**^	**Placebo Respimat®**^**a**^
	**(n = 146)**	**(n = 147)**	**(n = 146)**	**(n = 144)**
Patients with any adverse event	23 (15.8)	20 (13.6)	14 (9.6)	21 (14.6)
Patients with severe adverse events	2 (1.4)	0	0	0
Patients with serious adverse events^b^	2 (1.4)	0	0	0
Patients with investigator-defined drug-related adverse events	3 (2.1)	0	2 (1.4)	2 (1.4)
Patients with adverse events leading to discontinuation of study medication	1 (0.7)	0	0	0
**Most frequently reported adverse events (>1 patient on any treatment per treatment period)**				
Infections and infestations	9 (6.2)	4 (2.7)	6 (4.1)	7 (4.9)
Nasopharyngitis	5 (3.4)	1 (0.7)	2 (1.4)	2 (1.4)
Bronchitis	2 (1.4)	0	0	0
Oral candidiasis	0	0	1 (0.7)	2 (1.4)
Influenza	1 (0.7)	0	1 (0.7)	0
Rhinitis	0	0	1 (0.7)	1 (0.7)
Respiratory, thoracic and mediastinal disorders	5 (3.4)	8 (5.4)	4 (2.7)	7 (4.9)
Asthma exacerbation	4 (2.7)	3 (2.0)	1 (0.7)	5 (3.5)
Dyspnoea	0	3 (2.0)	0	1 (0.7)
Cough	0	1 (0.7)	2 (1.4)	0

^a^All patients in all groups on a background of maintenance treatment with stable medium-dose inhaled corticosteroids (400–800 μg budesonide or equivalent); ^b^Both patients experiencing serious adverse events required hospitalisation.

The most commonly reported adverse events by preferred term were asthma exacerbation and nasopharyngitis. In the tiotropium Respimat® 5 μg group, one patient reported dry mouth. No clinically relevant change in mean vital sign values was associated with tiotropium Respimat®.

## Discussion

In this dose-ranging study, tiotropium Respimat® was found to be an effective once-daily bronchodilator as add-on maintenance therapy in patients with symptomatic moderate asthma despite treatment with ICS. Once-daily doses of tiotropium Respimat® 5 μg, 2.5 μg or 1.25 μg, administered for 4 weeks per treatment, were each associated with statistically significant improvements in lung function compared with placebo Respimat®. Serious adverse events were rare and considered unrelated to treatment, and overall adverse-event incidence was comparable between all treatment groups.

All three doses of tiotropium Respimat® produced statistically significant improvements in the primary end point (peak FEV_1(0-3h)_ response) compared with placebo Respimat®. However, the response to 5 μg was larger than the responses to 2.5 μg and 1.25 μg, both of which were similar in magnitude. Further, in all other lung function parameters that were assessed, responses were consistently largest with tiotropium Respimat® 5 μg. Peak FEV_1(0-3h)_, FEV_1_ AUC_(0-3h)_, peak FVC_(0-3h)_ and FVC AUC_(0-3h)_ responses were statistically significantly greater with tiotropium Respimat® 5 μg versus either of the lower doses. The adverse-event profile for 5 μg was comparable with that for lower doses and placebo Respimat®.

In exploratory analyses, we observed a statistically significant reduction in ACQ-7 scores at the end of each 4-week treatment period with each of the three tiotropium Respimat® doses. However, the crossover design and the relatively short, 4-week, treatment periods are limitations with respect to drawing conclusions from the ACQ-7 score analysis.

The lung function and tolerability findings of the present trial are in accordance with those from previously published studies of tiotropium Respimat® as add-on to ICS ± LABA in patients with symptomatic asthma [10-12]. We await the results of two larger, replicate, Phase III, 24-week, randomised, double-blind trials in patients receiving moderate-dose ICS (NCT01172808 and NCT01172821).

## Conclusions

We have reported the first investigation of three different doses of tiotropium Respimat® (5 μg, 2.5 μg or 1.25 μg) in a patient population with symptomatic asthma treated with medium-dose ICS maintenance therapy. Tiotropium Respimat® 5 μg was found to be the most effective and consistent dose, with a safety profile comparable with that of placebo, thereby providing support for further investigation of tiotropium Respimat® in larger and longer-term Phase III trials in this population.

## Abbreviations

ACQ-7: Seven-question Asthma Control Questionnaire; AM2+®: Asthma Monitor2+ device; AUC: Area under the curve; CI: Confidence interval; FEV_1_: Forced expiratory volume in 1 second; FEV_1_ AUC_(0-3h)_: Forced expiratory volume in 1 second area under the curve measured within the first 3 hours after dosing; FVC: Forced vital capacity; FVC AUC_(0-3h)_: Forced vital capacity area under the curve measured within the first 3 hours after dosing; ICS: Inhaled corticosteroids; LABA: Long-acting β_2_-agonist; Peak FEV_1(0-3h)_: Peak forced expiratory volume in 1 second measured within the first 3 hours after dosing; Peak FVC_(0-3h)_: Peak forced vital capacity measured within the first 3 hours after dosing; PEF_am_: Morning peak expiratory flow; PEF_pm_: Evening peak expiratory flow; SE: Standard error; TALC: Tiotropium Bromide as an Alternative to Increased Inhaled Glucocorticoid in Patients Inadequately Controlled on a Lower Dose of Inhaled Corticosteroid.

## Competing interests

KMB has received compensation for organising or participating in advisory boards for Almirall Hermal, AstraZeneca, Boehringer Ingelheim, Cytos, Chiesi, Mundipharma, Novartis and Revotar Biopharmaceuticals, and in the past 3 years has participated as a speaker in scientific meetings or courses supported by Almirall Hermal, AstraZeneca, Boehringer Ingelheim, Novartis, Pfizer and Takeda. KMB has also received consulting fees from Ablynx, Apellis Pharmaceuticals, Chiesi and Cytos. The institution where KMB is employed has received compensation for the design, performance or participation in single- or multicentre clinical trials in the past 3 years from several companies, including Almirall, Boehringer Ingelheim, Cytos, GlaxoSmithKline, Mundipharma, Novartis, Pfizer, Revotar Biopharmaceuticals, Sterna AG and TEVA.

SK has received reimbursement for attending scientific conferences and fees for speaking or consulting from AstraZeneca, Boehringer Ingelheim, Chiesi, GlaxoSmithKline, MSD, Novartis and Nycomed. PMZ, AU, ZH and ME are employees of Boehringer Ingelheim. OA has received reimbursement for speaking from Boehringer Ingelheim, GlaxoSmithKline and Menarini.

## Authors’ contributions

KMB was the international coordinating investigator of the trial, contributed to the design of the study, recruited patients to the trial and contributed to the interpretation of data. PMZ, ME, ZH and AU each contributed to the study design, implementation, management and data analysis. SK and OA contributed to acquisition and interpretation of the data. All authors revised the article critically for intellectual content. All authors provided final approval of the article prior to submission.

## Supplementary Material

Additional file 1: Table S1 Adjusted mean differences in lung function and ACQ-7 score between tiotropium Respimat® and placebo Respimat®. Click here for file

## References

[B1] Global Initiative for Asthma Global strategy for asthma management and prevention. Revised 2014 [ http://www.ginasthma.org/local/uploads/files/GINA_Report_2014.pdf]

[B2] Chapman KR Boulet LP Rea RM Franssen E Suboptimal asthma control: prevalence, detection and consequences in general practice Eur Respir J 2008 31 320 325 10.1183/09031936.00039707 17959642

[B3] Partridge MR van der Molen T Myrseth SE Busse WW Attitudes and actions of asthma patients on regular maintenance therapy: the INSPIRE study BMC Pulm Med 2006 6 13 10.1186/1471-2466-6-13 16772035 PMC1483837

[B4] Rabe KF Adachi M Lai CK Soriano JB Vermeire PA Weiss KB Weiss ST Worldwide severity and control of asthma in children and adults: the global asthma insights and reality surveys J Allergy Clin Immunol 2004 114 40 47 10.1016/j.jaci.2004.04.042 15241342

[B5] Bateman ED Boushey HA Bousquet J Busse WW Clark TJH Pauwels RA Pedersen SE for the GOAL Investigators Group Can guideline-defined asthma control be achieved? The Gaining Optimal Asthma ControL study Am J Respir Crit Care Med 2004 170 836 844 10.1164/rccm.200401-033OC 15256389

[B6] Chowdhury BA Dal Pan G The FDA and safe use of long-acting beta-agonists in the treatment of asthma N Engl J Med 2010 362 1169 1171 10.1056/NEJMp1002074 20181964

[B7] Tashkin DP Celli B Senn S Burkhart D Kesten S Menjoge S Decramer M for the UPLIFT Study Investigators A 4-year trial of tiotropium in chronic obstructive pulmonary disease N Engl J Med 2008 359 1543 1554 10.1056/NEJMoa0805800 18836213

[B8] Vogelmeier C Hederer B Glaab T Schmidt H Rutten-van Mölken MPMH Beeh KM Rabe KF Fabbri LM for the POET-COPD Investigators Tiotropium versus salmeterol for the prevention of exacerbations of COPD N Engl J Med 2011 364 1093 1103 10.1056/NEJMoa1008378 21428765

[B9] Peters SP Kunselman SJ Icitovic N Moore WC Pascual R Ameredes BT Boushey HA Calhoun WJ Castro M Cherniack RM Craig T Denlinger L Engle LL DiMango EA Fahy JV Israel E Jarjour N Kazani SD Kraft M Lazarus SC Lemanske RF Jr Lugogo N Martin RJ Meyers DA Ramsdell J Sorkness CA Sutherland ER Szefler SJ Wasserman SI Walter MJ Wechsler ME Chinchilli VM Bleecker ER for the National Heart, Lung, and Blood Institute Asthma Clinical Research Network Tiotropium bromide step-up therapy for adults with uncontrolled asthma N Engl J Med 2010 363 1715 1726 10.1056/NEJMoa1008770 20979471 PMC3011177

[B10] Bateman ED Kornmann O Schmidt P Pivovarova A Engel M Fabbri LM Tiotropium is noninferior to salmeterol in maintaining improved lung function in B16-Arg/Arg patients with asthma J Allergy Clin Immunol 2011 128 315 322 10.1016/j.jaci.2011.06.004 21807250

[B11] Kerstjens HAM Disse B Schröder-Babo W Bantje TA Gahlemann M Sigmund R Engel M van Noord JA Tiotropium improves lung function in patients with severe uncontrolled asthma: a randomized controlled trial J Allergy Clin Immunol 2011 128 308 314 10.1016/j.jaci.2011.04.039 21636120

[B12] Kerstjens HAM Engel M Dahl R Paggiaro P Beck E Vandewalker M Sigmund R Seibold W Moroni-Zentgraf P Bateman ED Tiotropium in asthma poorly controlled with standard combination therapy N Engl J Med 2012 367 1198 1207 10.1056/NEJMoa1208606 22938706

[B13] Littner MR Ilowite JS Tashkin DP Friedman M Serby CW Menjoge SS Witek TJ Jr Long-acting bronchodilation with once-daily dosing of tiotropium (Spiriva) in stable chronic obstructive pulmonary disease Am J Respir Crit Care Med 2000 161 1136 1142 10.1164/ajrccm.161.4.9903044 10764302

[B14] van Noord JA Smeets JJ Custers FLJ Korducki L Cornelissen PJG Pharmacodynamic steady state of tiotropium in patients with chronic obstructive pulmonary disease Eur Respir J 2002 19 639 644 10.1183/09031936.02.00238002 11998992

