# Supplementary material for: Tiotropium Respimat® in asthma: a double-blind, randomised, dose-ranging study in adult patients with moderate asthma
Source: Respir Res. 2014 Jun 3;15(1):61. doi: 10.1186/1465-9921-15-61 (PMC4066691; doi:10.1186/1465-9921-15-61)
Supplement: Additional file 1: Table S1 — Adjusted mean differences in lung function and ACQ-7 score between tiotropium Respimat® and placebo Respimat®. [file 1465-9921-15-61-S1.doc]

**Additional file 1: Table S1 Adjusted mean differences in lung function and ACQ-7 score between tiotropium Respimat® and placebo Respimat®**

| **Treatment and parameter** | **Adjusted mean response^a^ (SE), mL** | **Difference from placebo** | |
| --- | --- | --- | --- |
|  |  | **Mean (95% CI), mL** | **P value** |
| **FEV_1_** | | | |
| FEV_1_ AUC_(0-3h)_ Tiotropium Respimat® 5 µg (n=143) Tiotropium Respimat® 2.5 µg (n=144) Tiotropium Respimat® 1.25 µg (n=144) Placebo (n=144) | 203 (27) 152 (27) 154 (27) 25 (27) | 178 (132, 224) 127 (81, 172) 129 (83, 175) | <0.0001 <0.0001 <0.0001 |
| **FVC** | | | |
| FVC AUC_(0-3h)_ Tiotropium Respimat® 5 µg (n=143) Tiotropium Respimat® 2.5 µg (n=144) Tiotropium Respimat® 1.25 µg (n=144) Placebo (n=144) | 110 (34) 47 (34) 36 (34) –28 (34) | 138 (86, 189) 75 (24, 126) 64 (13, 115) | <0.0001 0.0043 0.0149 |
| **ACQ-7** | | | |
| Tiotropium Respimat® 5 µg (n=143) Tiotropium Respimat® 2.5 µg (n=144) Tiotropium Respimat® 1.25 µg (n=144) Placebo (n=144) | 1.770 (0.061) 1.847 (0.061) 1.815 (0.061) 2.005 (0.061) | −0.235 (−0.343, −0.127) −0.158 (−0.265, −0.050) −0.190 (−0.298, −0.082) | <0.0001 0.0043 0.0006 |

^a^Response defined as change from study baseline (pre-treatment value measured at Visit 2 in the evening)
AUC, area under the curve; CI, confidence interval; FEV_1_, forced expiratory volume in 1 second; FEV_1_ AUC_(0-3h)_, forced expiratory volume in 1 second area under the curve measured within the first 3 hours after dosing; FVC, forced vital capacity; FVC AUC_(0-3h)_, forced vital capacity area under the curve measured within the first 3 hours after dosing; SE, standard error
